# A qualitative study of the reasons for low patient safety incident reporting among Indonesian nurses

**DOI:** 10.1590/0034-7167-2022-0583

**Published:** 2023-10-09

**Authors:** Bayu Anggileo Pramesona, Asep Sukohar, Surasak Taneepanichskul, M Fauzan Abdillah Rasyid

**Affiliations:** I Universitas Lampung. Bandar Lampung, Indonesia; II Chulalongkorn University. Bangkok, Thailand

**Keywords:** Patient Safety, Incident Reporting, Nurses, Qualitative Study, Indonesia, Segurança do Paciente, Relatórios de Incidentes, Enfermeiras, Estudo Qualitativo, Indonésia, Seguridad del Paciente, Informe de Incidentes, Enfermeras, Estudio Cualitativo, Indonesia

## Abstract

**Objectives:**

to investigate the reasons for low patient safety incident reporting among Indonesian nurses.

**Methods:**

this qualitative case study was conducted among 15 clinical nurses selected purposively from a public hospital in Lampung, Indonesia. Interview guidelines were used for data collection through face-to-face in-depth interviews in July 2022. The thematic approach was used to analyze the data.

**Results:**

in this present study, seven themes emerged (1) Understanding incident reporting; (2) The culture; (3) Consequences of reporting; (4) Socialization and training; (5) Facilities; (6) Feedback; and (7) Rewards and punishments.

**Final Considerations:**

these findings should be considered challenges for the patient safety committee and hospital management to increase patient safety incident reporting, particularly among nurses in the hospital.

## RESUMO

**Objetivos:**

investigar os motivos da baixa notificação de incidentes de segurança do paciente entre enfermeiros indonésios.

**Métodos:**

este estudo de caso qualitativo foi conduzido entre 15 enfermeiros clínicos selecionados intencionalmente de um hospital público em Lampung, Indonésia. Utilizou-se roteiro de entrevista para a coleta de dados por meio de entrevistas presenciais em profundidade em julho de 2022. A abordagem temática foi utilizada para análise dos dados.

**Resultados:**

neste estudo, emergiram sete temas: (1) Compreender a comunicação de incidentes; (2) A cultura; (3) Consequências da notificação; (4) Socialização e treinamento; (5) Instalações; (6) Comentários; e (7) Recompensas e punições.

**Considerações Finais:**

esses achados devem ser considerados desafios para o comitê de segurança do paciente e a gestão hospitalar para aumentar a notificação de incidentes de segurança do paciente, principalmente entre os enfermeiros do hospital.

## RESUMEN

**Objetivos:**

investigar las razones de la baja notificación de incidentes de seguridad del paciente entre las enfermeras de Indonesia.

**Métodos:**

este estudio de caso cualitativo se llevó a cabo entre 15 enfermeras clínicas seleccionadas intencionalmente de un hospital público en Lampung, Indonesia. Se utilizó un guión de entrevista para la recolección de datos a través de entrevistas presenciales en profundidad en julio de 2022. Se utilizó el enfoque temático para el análisis de datos.

**Resultados:**

en este estudio surgieron siete temas: (1) Comprender la notificación de incidentes; (2) La cultura; (3) Consecuencias de la notificación; (4) Socialización y capacitación; (5) Instalaciones; (6) Comentarios; y (7) Recompensas y Castigos.

**Consideraciones Finales:**

estos hallazgos deben ser considerados desafíos para el comité de seguridad del paciente y la gerencia del hospital para aumentar la notificación de incidentes de seguridad del paciente, especialmente entre las enfermeras del hospital.

## INTRODUCTION

According to the World Health Organization (WHO), a patient safety incident is an event that could have resulted, or did result, in unnecessary harm to a patient^(1)^. In order to reduce the number of unexpected events, hospitals are required to apply patient safety standards, which are implemented through incident reporting, analysis, and problem-solving^(2)^.

A type of surveillance known as a patient safety incident reporting system keeps track of, guards against, and lessens the frequency of patient safety incidents. It is part of the patient safety program that leads healthcare workers to improve patient safety in hospital settings^(3)^, and it has been developed in some countries worldwide^(4-5)^. Reporting patient safety incidents is crucial to enhancing patient safety^(6)^. Incident reporting results are used for decision-making and learning^(7)^. These systems rely on healthcare workers to report any incidents that jeopardize patient safety, allowing organizations and their employees to learn from others’ mistakes^(8)^. In this system, the role of nurses in improving patient safety is crucial.

The nursing process enables nurses to be physically near patients and spend more time with them. The continuous interaction between nurses and patients increases the risk of patient harm^(9)^. When a patient is injured, nurses must report the incident to identify and correct errors that endanger patient safety. The data gleaned from incident reports can be used to comprehend services’ scope and dangers and learn how to mitigate ongoing risks^(3)^. Reporting patient safety incidents should significantly reveal the risk of injury posed by nurses. An exemplary process for reporting safety incidents should emphasize ways to identify risks, clear risk priorities, methods for analyzing and investigating sources of risk, staff communication, and follow-up on arising issues^(3)^.

Several factors, including nurses’ reluctance to report for fear of punishment, lack of knowledge, greater emphasis on severe accidents^(10)^, inadequate reporting systems, management behavior, unclear definition of incidents, and fear of lawsuits, prevent the reporting process for patient safety from being optimally carried out^(11)^. Other studies have found that some factors like gender, marital status^(12)^, patient safety culture, nurses’ attitudes^(13)^, lack of government support, and political will^(14)^ can also influence patient safety incident reporting.

Patient safety incidents are still underreported in some countries. According to a study conducted in six ASEAN (Association of Southeast Asian Nations) countries, the lack of data on medical errors from nearly 50% of Southeast Asian countries demonstrates the region’s reporting system’s weakness^(15)^. A patient safety incident reporting system has been used in Indonesian healthcare facilities since 2006; however, its use has been limited due to a need for more understanding and clarity among Indonesian health workers^(16)^. Furthermore, there are still few reports of patient safety on a national level. The percentage of patient safety incident reports from 2015 to 2019 was 12%^(17)^. Another study demonstrates that Indonesia’s incident reporting system remains untimely^(18)^. Inadequate patient safety incident reporting can harm patient care^(19)^ and mortality quality^(20)^.

In order to fully understand the conditions experienced by nurses concerning reporting as a whole, it is necessary to make an effort to identify incident reporting problems from nurses’ perspective. These problems are related to a variety of safety incidents that occur in nursing services. Studies on nurses reporting safety incidents are still rare in Indonesia. Some previous quantitative studies have primarily provided information based on statistical findings^(13,21-22)^. Additionally, the studies that are currently available are limited to examining the effects of the workplace environment and cultural and practical barriers among healthcare workers (HCWs)^(23-24)^, nurses’ attitude towards reporting safety incidents^(25)^, and nurses’ experience in reporting safety incidents^(26)^.

## OBJECTIVES

To investigate the reasons for low patient safety incident reporting among Indonesian nurses.

## METHODS

### Ethical aspects

The Health Research Ethics Committee, Faculty of Medicine, Universitas Lampung, Indonesia, has granted ethical approval for this study. Furthermore, institutional approval was obtained from the hospital serving as the study site. Prior to the interview, verbal and written informed consent was obtained from all participants.

### Theoretical-methodological framework

This present study sought to investigate the critical reasons for low patient safety incident reporting from clinical nurses’ perspective, using Herzberg’s two-factor content theory of motivation^(27)^. Herzberg’s preliminary research yielded two distinct conclusions. An initial set of extrinsic conditions exists, including compensation, status, and working conditions. Then, there is an intrinsic set of conditions, such as a sense of accomplishment, increased responsibility, and acknowledgment. The two-factor content theory of motivation was pertinent to gaining a better understanding to achieve the current study’s purpose.

### Type of study

This study used a qualitative case study design that complied with the Consolidated criteria for reporting qualitative research (COREQ) to increase the research rigor and quality^(28)^. The qualitative data analysis technique used was Content Analysis in thematic mode^(29)^.

### Methodological procedures

#### Study setting and period

This study was conducted in July 2022 at a public district referral hospital in Lampung, Indonesia. The hospital chosen on purpose was accredited by the Hospital Accreditation Commission in 2019 and required a functional incident reporting system (internal and external reporting) managed by the hospital patient safety team; however, no incidents were reported internally and externally for the previous three years.

#### Data source

A total of 15 clinical nurses were purposively chosen for interviews based on their availability and willingness to participate using publicly available information about incident reporting. Participants were chosen based on their experience as registered nurses providing direct care to patients in inpatient, outpatient, operating room, and emergency departments.

The head nurses of the rooms helped researchers contact their nurses by providing their names and telephone numbers. Participants were contacted via telephone, and the head nurses also reached out to some nurses to recruit them for the study. As soon as they accepted the invitation, face-to-face interviews were scheduled. Nurses who refused to participate were excluded from the study. None, however, declined to participate in this study.

#### Data collection and organization

Developed semi-structured interviews with open-ended questions were used to achieve the purpose of this study. Before the interview, the protocol was sent to participants. After obtaining both written and verbal consent, the initial interviews were conducted at the participants’ work unit. Face-to-face in-depth interviews were conducted in Bahasa Indonesia, lasted between 50 and 60 minutes, and were voice-recorded. Notes were taken during the interviews to supplement the interview scripts. Data retrieval was stopped when saturation was reached because no new information could be found. There was no rise in the number of participants. Participant information is provided in Table 1.

**Table 1:** Participants’ characteristics (N=15)

Code	Age	Gender	Education	Work tenure	Work unit
P1	24	Female	Diploma	2 years	Outpatient department
P2	31	Female	Bachelor	7 years	Inpatient department
P3	36	Male	Bachelor	6 years	Outpatient department
P4	35	Female	Bachelor	11 years	Inpatient department
P5	37	Female	Bachelor	13 years	Emergency department
P6	47	Female	Diploma	22 years	Inpatient department
P7	27	Male	Bachelor	4 years	Operating room
P8	32	Female	Bachelor	7 years	Inpatient department
P9	51	Female	Diploma	28 years	Outpatient department
P10	33	Male	Diploma	6 years	Outpatient department
P11	28	Female	Diploma	6 years	Inpatient department
P12	26	Male	Bachelor	3 years	Inpatient department
P13	42	Female	Bachelor	15 years	Operating room
P14	39	Female	Diploma	15 years	Emergency department
P15	45	Female	Bachelor	18 years	Inpatient department

P - participants.

#### Data analysis

The recorded interviews were transcribed, translated, and checked for accuracy by a bi-lingual third party. The transcripts were not returned to the participants, and there was no feedback. Thematic analysis was used to synthesize and cross-reference emerging topics^(30)^. The following were the stages: 1) familiarization with the data; 2) generation of the initial codes; 3) theme search; 4) theme review; 5) themes definition and naming; and 6) report production^(31)^. Using the triangulation principle, we validated the data by examining it from the perspectives of nurses from different work units, through various lenses, and with various questions.

## RESULTS

There were 15 clinical nurses, with the majority being female, between 24-51 years old, holding a bachelor’s degree, having been working for less than ten years, and working in the inpatient department (Table 1). In our study, seven themes emerged as the causes of low patient safety incident reporting, including: (1) Understanding incident reporting; (2) The culture; (3) Consequences of reporting; (4) Socialization and training; (5) Facilities; (6) Feedback; and (7) Rewards and punishments. In this study, we classified participants’ responses based on emerging themes and summarized them in Table 2.

**Table 2:** Emerged themes according to participants’ responses

Themes	Participants’ responses	Quotes
Understanding incident reporting	Less understanding of the importance of incident reporting, its procedures, and contents	Q1: *Nurses have a poor understanding of incident reporting, such as what should be filled out on forms, the types of incidents, and when we should report the incidents.* (P2) Q2: *I did not see any urgency in reporting the incidents, and I also did not know where to report it if it happened.* (P4)
The culture	Afraid of being blamed by others The hospital reporting culture has not been implemented effectively	Q3: *Mostly because we are afraid of being blamed by others.* (R6) Q4: *When we reported the incidents, the hospital management did not emphasize the no-blame culture.* (P9) Q5: *I knew I would be embarrassed if I reported the incidents caused by my omission, so I just kept quiet.* (P4)
Consequences of reporting	Fear of legal action by patient or family Fear of being transferred to another hospital unit	Q6: *Fear of lawsuits by patients or their families when they realize we made an error, the case will grow larger and worsen.* (P1) Q7: *I am just afraid of being transferred to another unit if I report the incident caused by my omission, so I just keep quiet.* (P7)
Socialization and training	Inadequate socialization and training on incident reporting	Q8: *I have been here for more than three years and have never heard or received training on reporting patient safety incidents.* (P12) Q9: *How can we learn about incident reporting procedures if hospital leaders and the patient safety committee never provide information like socialization or incident reporting training?* (P8) Q10: *I am not sure what incidents should be reported because the patient safety committee only distributed information to the PIC in each unit. The PIC did not provide any additional information to the other staff.* (P11)
Facilities	There were insufficient resources to facilitate incident reporting.	Q11: *In our unit, the forms were not always ready.* (P13) Q12: *I have never seen any forms for reporting incidents here.* (P3) Q13: *The forms should always be available; it would be preferable if the hospital administration or patient safety committee could provide an online reporting system.* (P10)
Feedback	Feedback is predominantly slow or even non-existent	Q14: *Sometimes we reported* [the incidents]*, but there was no response from hospital management, prolonged response!* (P3) Q15: *We did it, we reported it, but there has been no response!* (P14) Q16: *The response was prolonged, even though we had reported* [the incident]. (P15)
Rewards and punishments	There were no incentives or penalties	Q17: *There was no money, no incentive of any kind.* (P5) Q18: *Demands good performance but does not recognize rewards.* (P8) Q19: *Even though it is our duty, there will be less motivation if there is no reward as a form of support.* (P11) Q20: *There are no rewards or punishments, so there is no motivation.* (P4)

P - participants; Q - quotes.

### Understanding incident reporting

Most participants reported low patient safety incident reporting due to a lack of understanding of incident reporting procedures and contents, such as what types of incidents, when, and how they should be reported (quotes 1-2) (Table 2).

### The culture

Because the hospital’s reporting culture was not well implemented, nurses feared being blamed when reporting incidents, as some participants pointed out in quotes 3-5 (Table 2).

### Consequences of reporting

Fear of litigation by patient/family and being transferred to another unit were also cited for low reporting. Fear of lawsuits from patients or family members has kept nurses from reporting incidents due to their omission. Some participants stated in quotes 6-7 (Table 2).

### Socialization and training

Because of a lack of socialization and training in incident reporting, nurses were unaware of the benefits of reporting incidents, the types of incidents that must be reported, when to report, and how to report them. Furthermore, socialization was only provided to the person-in-charge (PIC) in each unit rather than to all hospital employees, according to some participants in quotes 8-10 (Table 2).

### Facilities

Moreover, nurses perceived insufficient facilities to support incident reporting. The necessary documents were not always available in every unit. Furthermore, paper-based documents still exist, so it took time to fill out forms and send them to the hospital’s patient safety committee. Several participants stated in quotes 11-13 (Table 2).

### Feedback

Feedback on reported incidents, whether from the patient safety committee or hospital management, was generally slow or non-existent. Nurses’ intentions to report incidents have decreased due to this condition, as they believe it is a waste of time. Some participants explained this in quotes 14-16 (Table 2).

### Rewards and punishments

There was no system of rewards or punishments to encourage nurses to report incidents, so they had no motivation to do so, according to some participants in quotes 17-20 (Table 2).

## DISCUSSION

Patient safety is defined as “a framework of organized activities that creates cultures, processes, procedures, behaviours, technologies and environments in health care that consistently and sustainably lower risks, reduce the occurrence of avoidable harm, make errors less likely and reduce the impact of harm when it does occur”^(32)^. In Indonesia, reporting patient safety incidents has been fraught with difficulties. Most of the highlighted factors or barriers to reporting incidents have been investigated in previous studies, both in Indonesia^(23,33)^ and globally^(5,10)^. Even after many years, patient safety incident reporting in Indonesia still works.

### Understanding incident reporting

Most nurses in this study had a poor understanding of the types of incidents, when, and how they should be reported. This finding was consistent with a previous study that found that despite knowing about a reporting system in their hospital, staff did not know how to access an incident form or what to do with it once completed^(34)^. A previous study in Indonesia with similar findings highlighted a lack of knowledge and understanding regarding incident reporting^(14)^. A previous study identified three crucial phases of incident reporting: awareness and knowledge of the system, the ability to recognize reportable incidents, and the capacity to overcome any reporting barriers^(35)^. Failure to finish the first stage of this process, which involves having awareness and knowledge of the system, led to a failure in reporting incidents in general.

A previous study on nurses’ need for safety knowledge found that the experience and skills of developing patient safety-related science are significant to a nurse’s correct behavior in preventing mistakes when providing patient services^(36)^. To achieve goals in incident reporting, leaders must address staff with educational interventions that actively transfer knowledge^(37)^. A specific incident reporting management and education system within a learning, supportive working environment are essential components for enhancing nurses’ intent to report incidents, which could significantly increase patient safety^(38)^.

### The culture

A shaming and blaming culture may exacerbate the reporting culture for patient safety incidents^(39)^. Fear of being blamed or punished has also been identified as a significant cultural barrier^(16,40)^. A positive culture is required to raise individuals’ awareness of incident reporting^(14)^. Therefore, improving patient safety culture may also improve nurses’ attitudes towards incident reporting^(13)^. Culturally, HCWs tend to avoid conflicts with others, including friends and superiors^(23)^, and as a result, they do not engage in any reporting practices. Therefore, healthcare organizations must develop a supportive, learning, and safety-focused culture to encourage nurses to report patient safety incidents^(38)^.

A study in Uganda found that more than two-thirds of 158 participants preferred a work environment free of blaming and shaming to encourage compliance with incident reporting^(39)^. An interactive incident reporting training would be highly beneficial in promoting a patient safety culture^(41)^. Furthermore, effective communication within an organization has the potential to overcome cultural barriers^(23)^.

When reporting patient safety incidents to their colleagues, nurses showed fear, embarrassment, and discomfort, according to another finding of this study. A South Korean study found that HCWs, including nurses, were embarrassed to report safety incidents due to a range of emotional reactions, including shame, guilt, and depression, as well as behavioral changes, including insomnia, avoidance, and career changes^(42)^. Nurses’ mental health must be taken seriously, and assistance must be made available so that every incident can be reported without pressure.

### Consequences of reporting

Litigation raises concerns and fears of license revocation and individual penalties to encourage them to report. The high risk of litigation in the healthcare sector, combined with elements of extraordinary effort, becomes a powerful motivator for reporting. According to a previous study, most HCWs identified fear and reluctance to report as cultural barriers^(23)^.

Individual perception is the most common, with the fear of a lawsuit leading to the revocation of the practice license being the most common^(39-40)^. Other perceptions that influence reporting include the belief that events that can cause harm but can be avoided are not incidents that must be reported and the belief that reporting is unimportant^(43)^. Individual behavior can be affected by perceptions of a situation and vice versa. When someone has a negative impression of something, he or she is less likely to want to engage in that behavior.

### Socialization and training

Socialization and training on incident reporting are required for all staff to reinforce what has been taught^(44)^, particularly for nurses, who comprise the majority of HCWs in hospitals. A previous study discovered that a lack of socialization or training was identified as a practical barrier to reporting incidents among Indonesian HCWs^(23)^. Reporting should be simple, non-bureaucratic, and devoid of hierarchy^(45)^. In this case, hospital leaders should provide educational interventions such as in-house training to achieve incident reporting targets^(37)^. It is essential to provide organization-wide training to develop a shared understanding of patient safety incidents and a new set of reporting standards. In addition to this, clinical instructors should incorporate incident-related education into staff training to raise nurses’ awareness of incidents’ value^(38)^.

In Uganda, 55.7% of participants felt that incident identification training was necessary, and 60.1% felt that written guidelines were required. Staff is exposed to educational materials on how to report incidents^(39)^. Several hospitals in Indonesia hold regular safety and incident reporting training sessions. They did not, however, produce any excellent results. Socialization in the system must extend to all hospital units^(23)^.

### Facilities

The findings of this study revealed that facilities do not provide adequate support for patient safety reporting. Previous research discovered that organizational strength significantly impacts hospital patient safety efforts via management support for safety^(46)^. The organization’s attention, including providing appropriate facilities, can help nurses report incidents more efficiently. Organizations must establish a solid system to ensure that patient safety is a top priority.

Many countries, including Indonesia, have developed national incident reporting tools^(5,7,17)^. However, the Indonesian patient safety incident reporting system appeared to be ineffective in comparison to the Taiwan Patient Safety Reporting and Malaysian systems because it was unable to collect sufficient national incident reporting data and lacked transparency, which prevented learning at the national level^(47)^. In a Norwegian hospital, not all staff had access to the system, which was not integrated into all units^(44)^.

### Feedback

Many participants were sceptical of hospital management leaders’ and the patient safety committee’s commitment to providing feedback on reported incidents. As a result of the lack of feedback from the managerial levels, the majority of nurses complain. Inadequate incident reporting can undoubtedly be attributed to a lack of feedback, as feedback provides individuals with information about their actions. A lack of feedback has been recognized as a weakness in incident reporting in the past^(16,37)^. In contrast, adequately administered feedback makes the necessary enhancements to incident reporting^(48)^.

A further factor contributing to the system’s success is the leader’s commitment to reporting patient safety incidents. A previous study found that patient safety incidents underreporting in Indonesia was primarily due to a lack of hospital-level leadership and feedback^(14)^. Moreover, Indonesia has done little to document the lessons learned from national patient safety incident reporting or its potential to enhance patient outcomes or care procedures^(16)^.

### Rewards and punishments

In our study, nurses reported that organizations should prioritize safety incident reporting by increasing compensation. Individuals and teams are rewarded for improving safety in a safety-conscious culture. The lack of rewards and recognition is another reason employees are unaware of incident reporting. Consequently, the staff makes no positive changes^(45)^. On the other hand, a randomized experimental study found that incentives impact motivation for safety reporting but are not used over the long term because salary increases to improve safety culture have a short-term impact^(49)^.

The organization must develop an effective strategy to enhance the ongoing safety incident reporting over time. In addition, a study carried out in tertiary hospitals in the Philippines discovered that hospitals must enhance their knowledge, abilities, and attitudes towards a sustainable patient safety culture through training programs, benchmarking, institutionalization, and accreditation to promote patient safety^(50)^.

### Study limitations

One limitation of the study is that the findings cannot be generalized. This study may not represent a larger group of nurses because it used a purposive sampling technique. As a result, future research can be carried out to investigate the experiences of nurse groups from a larger population.

### Contributions to the field of nursing, health or public policy

This study produced intriguing findings significant for understanding nurses’ perspectives on reporting patient safety incidents. Given nurses’ perceived reasons for low patient safety incident reporting, nursing managers must provide practical guidance for their subordinates to openly and actively report patient safety incidents. The study’s findings help healthcare organizations create a work environment encourages nurses to report patient safety incidents.

## FINAL CONSIDERATIONS

Lack of understanding of incident reporting, blaming culture, fear of lawsuits, lack of socialization and training, inadequate facilities, no feedback, and no rewards and punishments system to report the incidents were identified as reasons for low patient safety incident reporting in this study. In order to increase patient safety incident reporting, particularly among nurses in our study area, the patient safety committee and hospital management leaders should consider these findings as challenges. Service organizations must consider the best approach to create a safety culture in the hospital by developing appropriate regulations.
